# Characterization of the Aquaporin-9 Inhibitor RG100204 In Vitro and in *db/db* Mice

**DOI:** 10.3390/cells11193118

**Published:** 2022-10-04

**Authors:** Marilina Florio, Angelica Engfors, Patrizia Gena, Jessica Larsson, Alessandro Massaro, Stella Timpka, Martina Kvist Reimer, Per Kjellbom, Eric Beitz, Urban Johanson, Michael Rützler, Giuseppe Calamita

**Affiliations:** 1 Department of Biosciences, Biotechnologies and Environment, University of Bari Aldo Moro, 70125 Bari, Italy; 2 Division of Biochemistry and Structural Biology, Department of Chemistry, Lund University, 22100 Lund, Sweden; 3 Red Glead Discovery AB, Medicon Village, 22381 Lund, Sweden; 4 Department of Management, Finance and Technology, Libera Università Mediterranea (LUM) “Giuseppe Degennaro” LUM University, 70010 Casamassima, Italy; 5 LUM Enterprise Srl, S.S. 100-Km18, Parco il Baricentro, 70010 Bari, Italy; 6 Department of Pharmaceutical and Medicinal Chemistry, Pharmaceutical Institute, Christian-Albrechts-University of Kiel, Gutenbergstr. 76, 24118 Kiel, Germany; 7 Apoglyx AB, Medicon Village, 22381 Lund, Sweden

**Keywords:** membrane transport, aquaglyceroporins, metabolic homeostasis, energy balance, glycerol metabolism, gluconeogenesis, calcein quenching, *Pichia pastoris*, proteoliposomes, stopped-flow light scattering

## Abstract

Aquaporin-9 (AQP9) is a facilitator of glycerol and other small neutral solute transmembrane diffusion. Identification of specific inhibitors for aquaporin family proteins has been difficult, due to high sequence similarity between the 13 human isoforms, and due to the limited channel surface areas that permit inhibitor binding. The few AQP9 inhibitor molecules described to date were not suitable for in vivo experiments. We now describe the characterization of a new small molecule AQP9 inhibitor, RG100204 in cell-based calcein-quenching assays, and by stopped-flow light-scattering recordings of AQP9 permeability in proteoliposomes. Moreover, we investigated the effects of RG100204 on glycerol metabolism in mice. In cell-based assays, RG100204 blocked AQP9 water permeability and glycerol permeability with similar, high potency (~5 × 10^−8^ M). AQP9 channel blocking by RG100204 was confirmed in proteoliposomes. After oral gavage of *db/db* mice with RG100204, a dose-dependent elevation of plasma glycerol was observed. A blood glucose-lowering effect was not statistically significant. These experiments establish RG100204 as a direct blocker of the AQP9 channel, and suggest its use as an experimental tool for in vivo experiments on AQP9 function.

## 1. Introduction

Aquaporin-9 (AQP9) is a member of the major intrinsic protein family of small transmembrane channels and displays broad substrate specificity. It is permeable to water and several neutral solutes, including glycerol [1,2]. We have previously demonstrated the importance of AQP9 for glycerol gluconeogenesis in mouse hepatocytes [3]. Furthermore, gluconeogenesis in mice is increased during fasting, which correlated with increased AQP9 expression, rising plasma glycerol concentration as a result of increased lipolysis, and enhanced glycerol permeability of hepatocyte membranes [4,5]. Moreover, regulation of AQP9 expression is known to depend on insulin in mice and rats [6,7] and also on leptin, at least in mice [8]. AQP9 has multifaceted roles in health and disease, triggering strong interest for its potential as biomarker and drug target [9].

Unchallenged *Aqp9^−/−^* knockout mice are healthy and show few phenotypes. In a C57BL/6 genetic background the only known effect of *Aqp9* gene deletion is an elevation of plasma glycerol [10] that presumably reflects reduced uptake and metabolism of glycerol by hepatocytes. However, when these *Aqp9^−/−^* mice were crossed to homozygous, leptin receptor deficient (*Lepr^db^*) obese, diabetic mice (henceforth *db/db*) of a C57BKS genetic background, F2 generation *Aqp9^−/−^* mice displayed reduced blood glucose concentration during a postprandial state, i.e., 4 h removed from food [10]. Notably, the effect was specific to the mixed C57BL/6 × C57BKS genetic background, and was not observed in a homogenous C57BL/6 *db/db* background [11].

Identification of small-molecule aquaporin inhibitors as experimental tools, and for potential therapeutic applications has been a highly desired, but challenging task. Some debate remains about the fidelity of the small number of actually identified aquaporin inhibitors [12,13,14,15]. We have previously described a number of aquaporin inhibitors that preferentially or specifically block three of the four aquaporins that facilitate glycerol transport, i.e., AQP3, AQP7, and AQP9 [3,16,17]. However, previously described AQP9 inhibitors were not suitable as tools for in vivo experimental use [3]. Moreover, one argument challenging the fidelity of currently described aquaporin inhibitors is that inappropriate methods were utilized to characterize these molecules [12].

In the current manuscript we describe the inhibition properties of a novel AQP9 inhibitor, RG100204, utilizing human AQP9-expressing CHO cells. A similar characterization of mouse AQP9 inhibition has been published recently, including characterization of the inhibitor specificity [18]. Furthermore, we extend this characterization in proteoliposomes, and demonstrate that in vivo treatment of mice with oral RG100204 can mimic previously described phenotypes of *Aqp9* gene deletion.

## 2. Materials and Methods

### 2.1. RG100204

RG100204 has been described in patent US20190127360 as Example 29, along with synthesis route, yield, and LC-MS characterization [19].

### 2.2. CHO Cell Water and Glycerol Permeability Assays

Water permeability assays and AQP9-expressing CHO cells have been described previously [17]. Briefly, cells grown in 96-well plates were loaded with 2.5 µg/mL of Calcein-AM (Biotium, San Francisco, CA, USA), and Probenecid (5 mM) (Sigma-Aldrich, St. Louis, MO, USA), before washing 1× in wash buffer (0.8 mM MgSO_4_, 5 mM KCl, 1.8 mM CaCl, 25 mM Hepes·Na, 106.5 mM NaCl; pH 7.4) plus 5 mM probenecid, and incubation in wash buffer (for water permeability assays), or wash buffer containing 500 mM glycerol (for glycerol permeability assays). Inhibitors were added to the respective incubation buffer in 1% DMSO. Cell shrinking was initiated by adding wash buffer containing 500 mM sucrose, 1% DMSO. Fluorescence-quenching time courses were recorded on a Fluostar Optima plate reader (BMG LABTECH, Ortenberg, Germany), Optima software version 2.20, and fit to a one-phase exponential decay function in GraphPad Prism 5 to obtain *K*% Permeability was calculated as:

$\%\mathrm{Permeability}=\left(1-\frac{K\;\left[\mathrm{AQP}9\;\mathrm{control}]-K\;[\mathrm{AQP}9\;\mathrm{inhibitor}\right]}{K\;\left[\mathrm{AQP}9\;\mathrm{control}\right]\;–K\;\left[\mathrm{CHO}\right]}\right)\times 100$

Whereby *K* corresponds to the rate constant, obtained from fitting the fluorescence intensity recordings after sucrose and glycerol addition, respectively, to a one-phase exponential function. [AQP9 control] describes AQP9-expressing cells incubated in 1% DMSO (Ducheva Biochemie, Haarlem, Netherlands), the inhibitor vehicle [AQP9 inhibitor] describes cells incubated at respective inhibitor concentration, and [CHO] describes recordings from CHO control cells that do not express ectopic AQP9 [3].

### 2.3. Production and Purification of 10 × His-hAQP9 Protein

High-expression clones of a recombinant human AQP9 bearing a 10-histidine tag fused at its N-terminus (*10 × His-hAQP9*) as well as a codon-optimized version of the construct were identified in *Pichia pastoris*. Prior to purification by Ni^2+^-NTA affinity chromatography, *n*-decyl-*β*-D-maltopiranoside (DM) was used to solubilize the protein from isolated membranes. For details see Supplemental Information and Figures S1–S5.

### 2.4. Liposome Preparation and 10 × His-hAQP9 Reconstitution

Purified *10 × His-hAQP9* protein was reconstituted into unilamellar liposomes following a previously described optimized protocol [20]. Briefly, chloroform was evaporated from 1 mL of 25 mg/mL of *Escherichia coli* polar lipids (Avanti Polar Lipids, Alabaster, AL, USA) under a nitrogen stream while rotating and drying under vacuum for 4 h at room temperature (RT). The resulting thin lipid film was hydrated in 1 mL of reconstitution buffer (20 mM Tris, 50 mM NaCl, pH 8.0) containing 2 mM ß-mercaptoethanol and incubated for 2 h, at RT. The suspension was then vortex-mixed and incubated on ice for 10 min. Aliquots of 200 µL (5 mg/mL lipids) were shock-frozen in liquid nitrogen and thawed on ice for 2 h before sonication in a bath sonicator for 120 min to obtain multilamellar liposomes. A reconstitution mixture was prepared at RT by sequentially adding the purified 10×His-hAQP9 protein (100 µg/mL; 1:50 phenyl-hexyl protein-lipid ratio), DM (1.8 mM) and liposome buffer (to a final volume of 1 mL). The 10×His-hAQP9 protein was omitted for empty liposomes. The mixture was vortex mixed, incubated on ice for 30 min and slowly injected through a 23-gauge needle into 22 mL of liposome buffer to dilute the detergent and force the protein to reconstitute into proteoliposomes. Multilamellar proteoliposomes/empty liposomes were collected by centrifugation for 1 h at 100,736× *g* at 4 °C. The supernatant was discarded and the resulting pellet resuspended in 1.5 mL of liposome buffer. The diameter of the liposome specimens was determined by electron microscopy. Briefly, pellets of proteoliposomes or empty liposomes were fixed in a mixture of 3% paraformaldehyde and 1% glutaraldehyde in 100 mM PBS at pH 7.4 for 4 h, at 4 °C. Specimens were post-fixed in 1% OsO_4_ in PBS for 60 min, at 4 °C. Fixed specimens were dehydrated in ethanol and then embedded in Epon (TAAB, Reading, England). Ultrathin sections were mounted on Cu/Rh mesh grids. The sections were stained with uranyl acetate and lead citrate and observed with a Zeiss EM 109 electron microscope (Zeiss, Oberkochen, Germany). Ten to fifteen micrographs were made from each gravitational pellet of liposome specimen and mean diameter was measured over an overall micrograph surface of about 2000 µm^2^. The diameter of the prepared liposome specimens was also measured using an N5 Submicron Particle Size Analyzer (Beckman Coulter Inc., Palo Alto, CA, USA). Before the stopped-flow light-scattering analysis (see below) samples were passed through a 27-gauge needle.

### 2.5. Proteoliposome–Glycerol Permeability Measurement

For measuring glycerol permeability of 10×His-hAQP9 proteoliposomes and empty liposomes, respectively, scattered light intensity time courses reflecting liposome volume change were recorded at 20 °C, 530 nm using an SFM-20 stopped-flow reaction analyzer (BioLogic, Claix, France) with a dead time of 1.6 ms and 99% mixing efficiency in <1 ms. Glycerol permeability was measured, as described previously [20,21], by mixing proteoliposomes in a hypotonic buffer (20 mM Tris-HCl, pH 8.0, made 220 mOsm with mannitol) with the same buffer plus added glycerol to generate a 150 mM glycerol gradient. Buffer osmolarities were measured by a vapor-pressure osmometer (Vapro Vapor Pressure Osmometer, EliTech, Puteaux, France). The glycerol permeability coefficient (*P*_gly_, cm/s), was calculated as:

$$P_{\mathrm{gly}}=\frac{V}{S\times \tau }$$

where *S* and *V* denote liposome surface and volume, and τ is the exponential time constant fitted to the liposome swelling phase, corresponding to glycerol entry, of the light- scattering time course. Inhibitors and respective vehicles (0.7 mM phloretin, 0.1% ethanol, 25 µM RG100204, 1% DMSO) were incubated with liposomes/proteoliposomes for 10 min before stopped-flow light-scattering recordings.

### 2.6. In Vivo Plasma Glucose and Glycerol Measurement

RG100204 was formulated in tetraethylene glycol (TEG; Sigma-Aldrich, St. Louis, MO, USA) at 5, 10, and 20 mg/mL, respectively. At t = 0, four groups of 10 mice were removed from food, followed by oral gavage at 2.5 mL/kg with the respective RG100204 formulation, or TEG (0 mg/mL group). Mice were kept fasting throughout the experimental period. Blood samples were collected from the tail vein of restrained animals at 90, 180, 270, and 360 min post-dosing. Blood glucose was measured using a handheld glucometer (ACCUCHEK AVIVA, Roche, Basel, Switzerland), and documented manually. Plasma (10 min centrifugation at 3000× *g*, 4 °C) was prepared within 45 min from blood collected in Li-heparin tubes and stored on ice until processing. Plasma samples were stored frozen at −20 °C before analysis, using the Free Glycerol Colorimetric/Fluorometric Assay Kit (Biovision, Milpitas, CA, USA), as described previously [3].

For RG100204 plasma concentration analysis, 20–25 µL Li-heparin blood per time point was frozen on dry ice immediately after collection and then stored at −20 °C until LC-MS analysis.

### 2.7. LC–MS Analysis

Calibration standards and QCs were prepared by spiking 20 µL of drug-free blank blood with 2.4 µL of RG100204 dissolved in DMSO at 200 µg/mL. Unknown samples, zero samples, and blanks were spiked with 2.4 µL DMSO. A volume of 40 µL acetonitrile containing griseofulvin (600 ng/mL) was added to each calibration standard, QC, zero sample and unknown sample, while 40 µL acetonitrile was added to blanks. Samples were mixed and centrifuged for 10 min at 6000× *g* at 20 °C. The supernatant was diluted 1:1 with water, and a 3 µL aliquot was subjected to LC-MS. The HPLC system consisted of a Dionex UltiMate 3000 RS pump, a Dionex UltiMate 3000 RS column compartment, and an AS Open auto-sampler (ThermoFisher Scientific, Waltham, MA, USA). Mass spectrometry was performed on a Q Exactive Plus (Orbitrap) accurate mass spectrometer equipped with a heated electrospray (H-ESI) interface (ThermoFisher Scientific) connected to a PC running the standard software Xcalibur 4.0.27.19. Analytes were separated on a Kinetex Phenyl-Hexyl, 2.6 µm, 50 × 2.1 mm analytical column (Phenomenex, Torrance, CA, USA). The HPLC was performed in the gradient mode using acetonitrile as organic phase (A) and 10 mM ammonium acetate in water as aqueous phase (B). The pump flow rate was set to 600 µL/min. The HPLC gradient A:B was 0–0.1 min 5:95, 0.4–1.7 min 97:3, 1.8–2.5 min 5:95. A generic tune file was used as MS tune file. Diisooctyl phthalate (m/z 391.28429) was used as lock mass, the MS was operated in the positive full-scan mode. Accurate mass of the monitoring ions ±5 mDa was used for the test item and internal standard peak integration. Full MS-SIM analysis was applied with the m/z ranges. Analyzer settings were as follows: max. trap injection time, 80 ms, sheath gas 40, aux gas 10, sweep gas 2, spray voltage 3.8 kV, capillary temperature 350 °C, heater 350 °C.

In vivo dosing of RG100204, as well as plasma sampling, blood glucose, and MS bioanalysis was conducted by Pharmacelsus Gmbh, Saarbrücken, Germany.

### 2.8. Statistical Analyses

Dose responses were calculated after log transformation of compound concentrations and fitting to a sigmoidal log-inhibitor vs. response function with a standard hillslope of −1.

Stopped-flow recordings were performed at least in triplicate. All data resulted from at least four independent preparations and were expressed as mean ± SEM. Differences between experimental groups were examined for statistical significance using the Student’s *t* test, with *p* < 0.01 denoting presence of a statistically significant difference.

In vivo data were analyzed by 2-way analysis of variance (ANOVA), considering time points as repeated measures, followed by Bonferroni post-tests. RG100204 plasma concentration was analyzed by linear regression. Calculations and plots were prepared in GraphPad Prism 5.0 (GraphPad Software, San Diego, CA, USA). Data were expressed as means ± SD.

## 3. Results

### 3.1. Characterization of RG100204 Inhibition Properties on CHO Cells Expressing AQP9 and AQP9 Proteoliposomes

**Figure 1:**
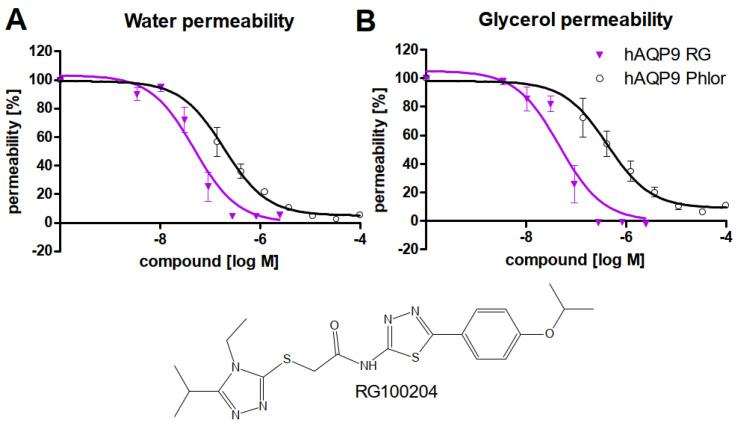
Inhibitor characterization in calcein-loaded CHO cells expressing human AQP9. Permeability in % was calculated in comparison to the parental CHO cell line, without ectopic AQP9-expression. (**A**) Dose-response of water permeability inhibition, (**B**) dose-response of glycerol permeability inhibition. Potency of blocking water permeability was similar for both substances, while RG100204 blocked glycerol permeability with higher efficacy. *n*=3, IC_50_ RG100204 (RG, magenta, filled triangles): water permeability, ~5 × 10^−8^ M (R^2^ = 0.94), glycerol permeability ~5 × 10^−8^ M (R^2^ = 0.96), IC_50_ Phloretin (phlor, black, open circles): water permeability, ~2 × 10^−7^ M (R^2^ = 0.92), glycerol permeability ~4 × 10^−7^ M, (R^2^ = 0.92).

In order to characterize the potency and efficacy of RG100204 for inhibition of AQP9 permeability, water permeability and glycerol permeability were measured in calcein-loaded CHO cells ectopically expressing human AQP9, as well as in control CHO cells, without AQP9 expression (Figure 1). Compared to the positive control phloretin, inhibition of AQP9 permeability by RG100204 was more potent (IC_50_ water permeability ~2 × 10^−7^ M vs. ~5 × 10^−8^ M, IC_50_ glycerol permeability ~4 × 10^−7^ M vs. ~5 × 10^−8^ M). Thus, inhibition of permeability by RG100204 was comparable for the two channel substrates, water and glycerol. Moreover, inhibition of glycerol permeability by RG100204 was more efficacious, compared to phloretin (remaining permeability −1.3% ± 0.4 vs. 16.2% ± 2.6).

**Figure 2:**
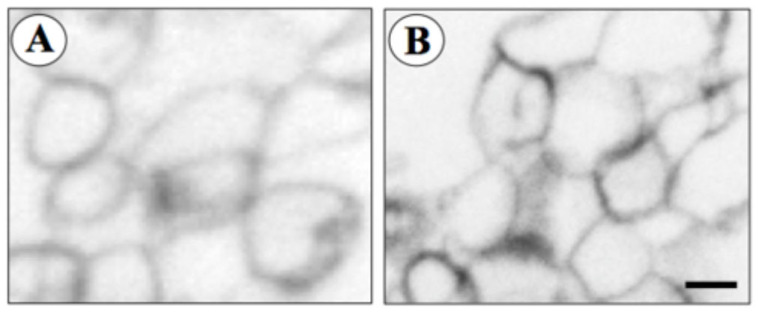
Electron microscopy analysis of gravitational pellets of the liposome specimens. Electron micrographs showing control empty liposome specimens (**A**) and 10×His-hAQP9 proteoliposome specimens (**B**) prepared as described in Materials and Methods. Bar, 100 nm.

In order to further support the fidelity of RG100204 as an AQP9 inhibitor, we chose to express human *AQP9* in the yeast *P. pastoris*, and to reconstitute the purified recombinant protein 10×His-hAQP9 into unilamellar liposomes prepared from *E.* *coli* polar lipids (Figures S1–S5). Successful preparation was initially confirmed by electron microscopy (Figure 2A,B) and particle size analysis, establishing mean diameters of the empty control liposomes and 10×His-hAQP9 proteoliposomes of 219 ± 18 nm and 229 ± 63 nm, respectively.

**Figure 3:**
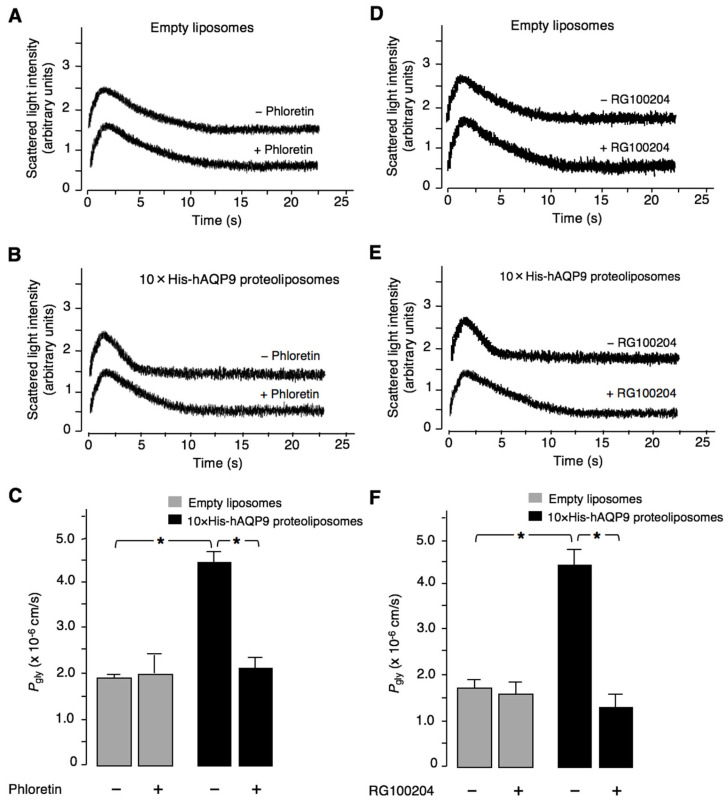
RG100204 characterization in human AQP9 proteoliposomes. Recombinant hAQP9 bearing a 10-histidine tag fused at its N-terminus (10×His-hAQP9) was incorporated into unilamellar liposomes and the glycerol permeability (*P*_gly_) was measured by stopped-flow light scattering in presence or absence of AQP9 inhibitor (0.7 mM phloretin or 25 µM RG100204) in comparison to the empty liposomes, with no AQP9 incorporation. (**A**) Representative light-scattering traces of empty liposomes in absence or presence of 0.7 mM phloretin, a non-selective inhibitor of facilitated transport of glycerol used as positive control, (**B**) representative light-scattering traces of hAQP9 proteoliposomes in absence or presence of phloretin, (**C**) graph showing proof of functionality of the reconstituted 10×His-hAQP9; the glycerol permeability of the proteoliposomes is significantly higher than that of control empty liposomes and is markedly decreased after treatment with phloretin. Representative light-scattering traces of empty liposomes (**D**) or hAQP9 proteoliposomes (**E**) in absence or presence of RG100204; and (**F**) illustrates that treatment with RG100204 induces a strong reduction of the permeability to glycerol mediated by AQP9 whereas no changes are seen in the empty liposomes. Data are mean ± SEM from four independent preparations. * *p* < 0.01.

Functionality of the AQP9 channel in the proteoliposomes was established by measuring the glycerol permeability coefficient (*P*_gly_; cm/s) of the proteoliposomes and control liposomes by the stopped-flow light-scattering method, after applying a 150 mM inwardly directed glycerol gradient. Figure 3 shows representative light-scattering traces, with the initial increase in light-scattering intensity resulting from osmotic water efflux (liposome shrinkage), followed by a slower decrease caused by glycerol influx, followed by a parallel osmotic entry of water (liposome swelling). No differences were observed in glycerol permeability (*P*_gly_) between empty liposomes treated with phloretin and vehicle-treated empty liposomes (1.38 ± 0.17 × 10^−6^ cm/s and 1.98 ± 0.39 × 10^−6^ cm/s, respectively; Figure 3A,C). By comparison, glycerol permeability of the proteoliposomes was significantly higher (4.42 ± 0.28 × 10^−6^ cm/s; *p* < 0.01; Figure 3B,C), thus indicating functional reconstitution of 10×His-hAQP9. The *P*_gly_ value of the proteoliposomes was significantly reduced by pre-treatment with the positive control inhibitor phloretin, applied at 0.7 mM (2.10 ± 0.21 × 10^−6^ cm/s, −52.5%; *p* < 0.01; Figure 3C). Similarly, empty liposomes were not affected by RG100204 compared to its 1% DMSO vehicle (Figure 3D,F), while 10×His-hAQP9-proteoliposomes treated with 25 µM RG100204 exhibited significantly lower *P*_gly_ compared with vehicle treated proteoliposomes (1.26 ± 0.33 × 10^−6^ cm/s and 4.43 ± 0.34 × 10^−6^ cm/s, respectively; −71.5%; *p* < 0.01; Figure 3E,F).

### 3.2. RG100204 Increases Plasma Glycerol in Fasted db/db Mice

**Figure 4:**
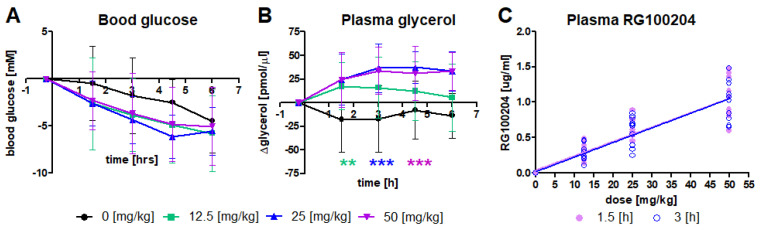
Effects of three different p.o. doses of RG100204 in C57BKS *db/db* mice. Mice were fasted throughout the experiment. A continuous fall in blood glucose was more pronounced in all three groups of RG100204-treated mice, but was not statistically significant (**A**). Plasma glycerol increased in all three inhibitor-treated groups, compared to controls (**B**). RG100204 plasma concentrations were quantified by LC-MS at 1.5 h and 3 h after dosing (**C**). Statistical analysis (**A**,**B**): 2-way ANOVA, considering time points as repeated measures, and Bonferroni post-tests. Highest post-test significance levels along the time course are indicated as asterisks for panel B, in colors matching the legend; ** *p* < 0.01, *** *p* < 0.001. (**C**) Linear regression analysis for each time point suggests a dose-dependent increase in plasma RG100204 concentration, slope = 0.02 (both time points), r^2^ = 0.82 (1.5 h) and 0.81 (3 h).

In order to investigate if RG100204 is suitable as a tool compound for studying AQP9 function in vivo, we tested previously observed effects of *Aqp9* gene deletion on blood glucose and plasma glycerol. Diabetic C57BKS *db/db* mice were exposed to three different oral doses of RG100204, while removing mice from food, to mimic conditions that were previously established in *Aqp9^-/-^ db/db* mice [10]. Blood glucose was monitored over a 6-h period in vehicle-treated and RG10024-treated groups of mice, whereby blood glucose concentration dropped in all four groups during fasting (Figure 4A). A faster decline in blood glucose that was observed in all three RG100204 treated groups did not reach statistical significance in 2-way ANOVA. The reduction in blood glucose was mirrored by an increase in plasma glycerol in the three RG100204-treated groups, but not in the vehicle-treated group (Figure 4B). Plasma bioanalysis revealed similar concentrations of RG100204 at two determined time points after oral dosing (1.5 h and 3 h), whereby increasing doses of RG100204 resulted in increased detected RG100204 plasma concentration (Figure 4C).

## 4. Discussion

In the current study, we describe the characterization of RG100204 as a novel, specific inhibitor of human aquaporin-9 water and glycerol permeability in a calcein-quenching- based cell assay and by stopped-flow light-scattering using hAQP9 proteoliposomes. We have previously described a short list of compounds with similar characteristics [3,17]. In general, few aquaporin inhibitors have been described to date, and the level of characterization of several described inhibitors has been criticized as insufficient [12,13,14]. One particular point of critique was that cell-based assays allowed for confounding influences that could result in incorrect identification of small molecules as AQP inhibitors. We thus sought to establish RG100204 as an inhibitor of human AQP9 independently, in a cell-free assay. AQP9 was therefore expressed and purified from *P. pastoris* before incorporation into artificial proteoliposomes. RG100204 strongly reduced glycerol permeability in this cell-free system, thereby corroborating RG100204 as an AQP9 inhibitor.

Furthermore, as the tested proteoliposomes essentially do not contain any other protein except for AQP9, which was purified by Ni^2+^-NTA affinity chromatography and verified by Western blotting (see Supplemental Information and related Figure S5), these assays provide concrete evidence for direct channel-blocking of AQP9 by RG100204, as opposed to other modes of action, e.g., by modifying surface expression, which might be observed in cell-based assays.

Moreover, previously identified AQP9 inhibitors were not suitable as tool compounds for in vivo experimental work in mouse models [3]. Much of the current knowledge on the physiological role of AQP9 has been derived from experiments conducted with *Aqp9^−/−^* mice [10,22]. While these mice have proven very useful in this area of research, it is possible that some of the effects of *Aqp9* deletion, e.g., the blood glycerol elevating effect in *Aqp9^−/−^* mice [10] result from developmental changes or chronic adaptions that are not linked to an acute reduction of AQP9-facilitated hepatocyte glycerol uptake and metabolism [3]. Moreover, reduced blood glucose levels in *Aqp9^−/−^* mice have been demonstrated in a mixed C57Bl6 × BKS genetic background [10], thus theoretically allowing that genetic linkage between *Aqp9* and a blood glucose modifier gene could have caused these observations. In the current study, *db/db* mice of the BKS background were used, which develops a severe diabetic phenotype, in contrast to *db/db* mice of the Bl6 background [23]. An observed average reduction in blood glucose by RG100204 treatment was not strong enough to reach statistical significance, but agrees with a direct blood glucose-modifying role of AQP9 via glycerol uptake and gluconeogenesis from this substrate.

## 5. Conclusions

Altogether, our results demonstrate functional inhibition of AQP9 by RG10204 both in vitro and in vivo. A dose-dependent elevation of *db/db* mouse plasma glycerol was seen after oral administration of the compound. A blood glucose-lowering effect was also observed, although it was not statistically significant. These experiments establish RG100204 as a direct blocker of the AQP9 channel, and suggest this heterocyclic compound as a novel tool for investigating the physiological and pathophysiological role of AQP9 in health and disease. These findings complement our recent demonstration of RG100204 blocking the role of AQP9 in sepsis [18,24].

## Data Availability

Raw data are available from the authors on request.

## Supplementary Materials

Supplementary material for this article can be found online at: https://www.mdpi.com/article/10.3390/cells11193118/s1 [25,26,27,28,29]. Figure S1: Screening of *P. pastoris* clones with the highest heterologous production of recombinant hAQP9; Figure S2: Time course of 10×His-hAQP9 expression; Figure S3: Crude membranes containing 10×His-hAQP9 washed with urea; Figure S4: detergent screening; Figure S5: Purification of the 10×His-hAQP9 protein.
