# Supplementary material for: Characterization of the Aquaporin-9 Inhibitor RG100204 In Vitro and in db/db Mice
Source: Cells. 2022 Oct 4;11(19):3118. doi: 10.3390/cells11193118 (PMC9562188; doi:10.3390/cells11193118)
Supplement: Supplementary file 1 [file cells-11-03118-s001.zip › cells-1922751-supplementary.pdf]

Supplemental Information

Production of 10×His-hAQP9 protein in P. pastoris yeast cells

The wild type (WT) coding sequence of human *AQP9* (885 bp) as well as a codon optimized version (CO) for *P. pastoris* obtained using GeneArt tool, were amplified with Phusion HF DNA polymerase. Specifically, the forward primer hAQP9co_FW_nhis TTGAATTCCGACC*GAAAACCTGTATTTTCAGGGC***ATG**CAACCAGAGGGTGCTGAG and the reverse primer hAQP9co_REV_nhis AAGCGGCCGC**TTA**CATGATAAC-GGACAACTCGTAC were used for the CO construct (restriction sites *Eco*RI and *Not*I underlined, encoded TEV site in italics, start and stop sites of *AQP9* in bold). The amplified sequence was cloned into the "FOR_TEV" plasmid, which is a modified pPICZB plasmid (EasySelect™, Invitrogen) encoding an N-terminal 10×His tag as described in the Supplementary Information in [25]. In the resulting construct the gene of interest is under control of the strong methanol inducible AOX1 promoter for high-level expression of the encoded hAQP9 with a TEV cleavable His tag. The integrity of the insert was confirmed by sequencing and the 4240 bp 10×His_TEV_pPICZB_hAQP9 plasmids were linearized using *Sac*I and transferred into electrocompetent X-33 *P. pastoris* cells according to EasySelect™ (Invitrogen). Zeocin selection of high expression clones and small scale expression were done as previously described [26]. Briefly, potential jackpot clones were screened for methanol induced expression, using immunoblotting (described below) to assess the extent of AQP9 production in crude lysates.

Overproduction was evaluated on small scale of 24 clones of each construct using an anti-His-tag antibody where 200 ng of His-tagged recombinant spinach AQP (SoPIP2;1) was used as positive control and standard [27]. As shown in Figure S1, where the highest expressing clones (CO2, CO8, CO17, WT4 and WT22) were selected for final comparison, CO8 resulted as the *P. pastoris* transformant clone with the highest production of 10×His-hAQP9 protein.

In order to obtain large amounts of protein, the 10×His-hAQP9 clone CO8 was selected for large scale protein production in a 3 L bench top fermenter (Belach Bioteknik, Stockholm, Sweden) essentially as described previously [28]. Briefly, cells were grown in a 150 mL BMGY culture o/n and added to the 1.5 L medium in the fermentor (1.5 L basal salt medium, 6.5 mL PTM salts). One liter of basal salts medium contained 26.7 mL 85% H_3PO_4 and 0.93 g $CaSO_4 \times 2H_2O$; 18.2 g $K_2SO_4 \times 7H2O$; 4.13 g KOH; 40 g glycerol and the pH was adjusted to 5.0 after sterilization by addition of 30% NH_4OH. PMT1 trace salts consisted of (in grams per liter): $CuSO_4 \times 5H_2O$, 6.0; NaI, 0.08; $MnSO_4 \times H_2O$, 3.0; $Na_2MoO_4 \times 2H_2O$, 0.2; H_3BO_3, 0.02; $CoCl_2$, 0.5; $ZnCl_2$, 20; $FeSO_4 \times 7H2O$, 65; biotin, 0.2; and concentrated sulfuric acid, 5 mL. Additional glycerol (100 mL 50% glycerol, 2.4 mL PTM1 salts) was added after 24 h and when this was consumed protein expression was induced by switching to a steady feed of methanol (100% MeOH, 1.2% PTM1 salts). Samples were collected at 0, 21, 42 and 46 hours of methanol induction and cells were finally harvested by centrifugation and stored at -80 ºC.

The time course of 10×His-hAQP9 production was assessed by Western blotting analysis of aliquots of the *P. pastoris* transformant culture collected at the different time points to define the best moment for harvesting the grown medium with the recombinant protein. The highest level of 10×His-hAQP9 protein overproduction was seen after 46 h of methanol induction (Figure S2). About 200 g of wet cells were harvested at this time point.

Isolation of membranes from P. pastoris yeast cells

Forty-five grams of cells were thawed while stirring for 30 minutes at 4 ºC in 200 mL ice-chilled breaking buffer (50 mM NaH_2PO_4, 1mM EDTA, 5% glycerol, 1 mM PMSF, pH 7.4). An equal volume of chilled and acid-washed glass beads (0.5 mm diameter) was added to the suspension. The bead-beater device (BeadBeater, BioSpec Products,

Bartlesville, OK) was filled with cell suspension and cells were lysed by running the bead-beater 15 × 30 seconds with 30 seconds pause between each run. The cell suspension without beads was transferred into the French press device and cells were once more lysed at 10,000 psi (~1 kbar). The lysate was transferred to centrifuge bottles and large cell debris and unbroken cells were removed by low-speed centrifugation in JA-14 Fixed-Angle Aluminum Rotor (Beckman) for 30 minutes at 26,500 × g at 4 ºC. The resulting supernatants containing the cell contents were subsequently ultra-centrifuged in Type 70Ti fixed-angle titanium rotor (Beckman) for 1 h at 168,128 × g at 4 ºC. The supernatant was discarded and the pellet was homogenized using a potter homogenizer in 15 mL urea buffer (4 M urea, 5 mM Tris-HCl, 2 mM EDTA, 2 mM EGTA, pH 9.5). Crude membranes were poured into centrifuge tubes, incubated on ice for 20 min and centrifuged in Type 70Ti Fixed-Angle Titanium Rotor (Beckman) for 90 min at 168,128 × g at 4 ºC. The resulting supernatant was discarded and the pellet was homogenized in 15 mL of buffer A (20 mM HEPES-NaOH, 50 mM NaCl, 10% glycerol, 2 mM ß-mercaptoethanol, pH 7.8). The membranes stripped of peripheral membrane proteins were poured into the tubes and centrifuged for 90 min at 168,128 × g at 4 ºC. The supernatant was discarded and the pellet (washed membranes) was homogenized in 10 mL buffer A. Finally, the efficacy of the cellular lysis was evaluated by determining the total protein concentration by the Bearden assay. The extent of protein expression was determined by immunoblotting as described below.

Western blotting

Protein samples were loaded together with PageRuler Plus Prestained Protein Ladder (Thermo Fisher Scientific) and 200 ng purified SoPIP2;1 [27,29] as positive control on precast 12% SDS–PAGE gels (Bio-Rad Laboratories). The gels were run on 180 V under reducing conditions and transferred to PVDF membranes (Bio-Rad) using Trans-Blot turbo (Bio-Rad). The membranes were activated in methanol, rinsed, and blocked (3% BSA in 1×TBS) for 1 h at RT followed by incubation with primary antibody (QIAGEN mouse Tetra-His, 1:2000 dilution in 3% BSA, 1×TBS, 0.05% NaN_3) o/n at 4 ºC with constant shaking. After washing (3 × 10 min in 1×TBS, 0.1% Tween-20), the membranes were incubated with the secondary antibody (Amersham Anti-mouse-HRP 1:10 000 in 1×TBS, 10% dry milk) for 1 h and then washed as above before analysis of protein detection.

Detergent screening

Crude membranes from cell lysate prepared from large scale production (about 45 g of wet cells) of high-yielding CO8 clone were stripped by urea wash (Figure S3) before moving on to the extraction and solubilization of 10×His-hAQP9. Twelve small aliquots of homogenized washed membranes were diluted in buffer A and an equal volume of 12 different detergents, were added slowly to the membrane while stirring. The selected mild and anionic detergents [FOS-choline-12 (F-12), Dimethyldecylphosphine oxide (APO-10), lauryldimethylamine N-oxide (LDAO), n-Tetradecyl-N,N- dimethylamine-N-oxide (TDAO), maltose-neopentyl glycol (MNG), 3-[(3-cholamidopropyl) dimethylam-monio] -1-propanesulfonate (CHAPS), 5-Cyclohexyl-1-penty l-β-d-maltoside (CY-5), n-decyl-ß-D-maltopyranoside (DM), n-Dodecyl-β-maltoside (DDM), n-nonyl-β-d-glucopyranoside (NG), Octyl-β-glucopyranoside (OG), n-Octyl-1-thio-β-D-glucopyranoside (OTG)], were prepared in buffer A with a concentration of 20 times higher than their CMC. The solubilized solution was incubated on stirring for 1 h at RT. The unsolubilized material was spun down in a 70Ti Fixed-Angle Titanium Rotor (Beckman) for 30 min at 168,128 × g at 4ºC. The supernatant with the solubilized protein and the corresponding pellet, were submitted to SDS PAGE followed by Coomassie blue staining and immunoblotting to assess the extent of detergent suitability (Figure S4). The mild and non-ionic detergent *n*-decyl-*β*-D-maltopiranoside (DM) at a final concentration 10 times higher than its CMC (18 mM) turned out to best fit this criterion and was hence chosen for subsequent purification.

Extraction of 10×His-hAQP9

A volume of 5 mL of homogenized washed membranes were diluted in 15 mL of buffer A. An equal volume (20 mL) of the mild and anionic detergent DM with a concentration 20 times higher than its CMC was prepared in buffer A and added slowly to the washed membranes, while stirring. The solubilized solution was incubated on stirring for 1 h at RT. The unsolved material was spun down in a 70Ti Fixed-Angle Titanium Rotor (Beckman) for 30 minutes at 168,128 × g at 4 ºC and the supernatant containing the 10×His-hAQP9 solubilized protein, was submitted to Ni-affinity purification.

Purification of 10×His-hAQP9 by Ni^{2+}-NTA affinity chromatography

The DM-solubilized 10×His-hAQP9 protein was purified to near homogeneity by immobilized metal-chelated (nickel-nitrilotriaceticacid, Ni^{2+}-NTA) affinity chromatography. Briefly, 40 mL of solubilized 10×His-hAQP9 protein were mixed with 500 µL of resuspended Ni^{2+} loaded NTA agarose (Qiagen, Valencia, CA) in 1 mL of buffer B (20 mM HEPES-NaOH, 300 mM NaCl, 10% glycerol, 2 mM ß-mercaptoethanol, 0.4% DM, pH 7.8). The mixture was incubated overnight at 4 ºC on a rolling device. Then, the mixture was poured into an empty Poly-Prep chromatography column (Bio-Rad. Hercules, CA) and washed 10 times at gravity flow rate with 3.0 mL of buffer B added with 80 mM imidazole. The bound 10×His-hAQP9 protein was eluted with 1.5 mL buffer B supplemented with increasing concentrations of imidazole (40, 60, 80, 100, 200, 300 and 500 mM). Binding of 10×His-hAQP9 to the Ni-NTA column was relatively strong in 36 mM DM as no elution was seen below 100 mM imidazole (Figure S5). Most of the protein was eluted with imidazole concentrations of 200 and 300 mM. By immunoblotting 10×His-hAQP9 was detected as a band of 34.5 kDa likely corresponding to the monomeric form of the protein and two additional bands of minor intensity and higher sizes probably representing the oligomeric forms (Figure S5). Starting with 20 g of wet cells, about 8 mg of pure 10×His-hAQP9 were eluted from the Ni^{2+}-NTA agarose beads giving a total yield of about 0.4 mg of pure 10×His-hAQP9 protein per gram wet weight cells. Fractions eluted at 200 mM and 300 mM imidazole were pooled and diafiltrated, using 30-K Amicon Ultra-4 centrifugal filter device (Millipore, Cork, Ireland), to remove the imidazole and simultaneously concentrate the protein at final concentrations of about 2 mg/mL.

References

25. Kirscht, A.; Kaptan, S.S.; Bienert, G.P.; Chaumont, F.; Nissen, P.; de Groot, B.L.; Kjellbom, P.; Gourdon, P.; Johanson, U. Crystal Structure of an Ammonia-Permeable Aquaporin. *PLoS biology* **2016**, *14*, e1002411, doi:10.1371/journal.pbio.1002411.
26. Norden, K.; Agemark, M.; Danielson, J.A.; Alexandersson, E.; Kjellbom, P.; Johanson, U. Increasing gene dosage greatly enhances recombinant expression of aquaporins in Pichia pastoris. *BMC biotechnology* **2011**, *11*, 47, doi:10.1186/1472-6750-11-47.
27. Karlsson, M.; Fotiadis, D.; Sjovall, S.; Johansson, I.; Hedfalk, K.; Engel, A.; Kjellbom, P. Reconstitution of water channel function of an aquaporin overexpressed and purified from Pichia pastoris. *FEBS Lett* **2003**, *537*, 68-72.
28. Ampah-Korsah, H.; Anderberg, H.I.; Engfors, A.; Kirscht, A.; Norden, K.; Kjellstrom, S.; Kjellbom, P.; Johanson, U. The Aquaporin Splice Variant NbXIP1;1alpha Is Permeable to Boric Acid and Is Phosphorylated in the N-terminal Domain. *Frontiers in plant science* **2016**, *7*, 862, doi:10.3389/fpls.2016.00862.
29. Tornroth-Horsefield, S.; Wang, Y.; Hedfalk, K.; Johanson, U.; Karlsson, M.; Tajkhorshid, E.; Neutze, R.; Kjellbom, P. Structural mechanism of plant aquaporin gating. *Nature* **2006**, *439*, 688-694, doi:10.1038/nature04316.

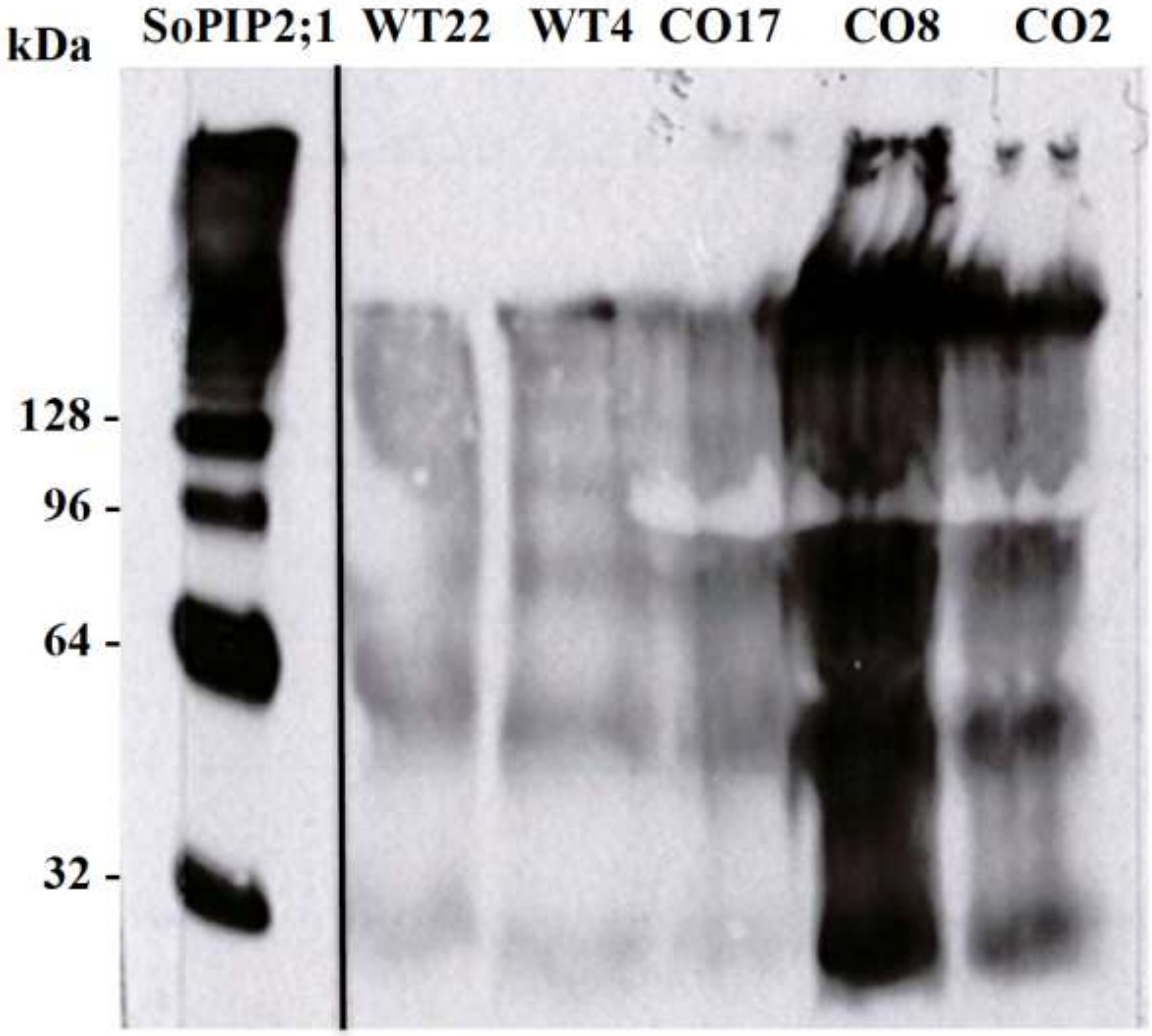

Figure S1. Screening of P. pastoris clones with the highest heterologous production of recombinant hAQP9. Western blot showing relative expression levels of recombinant 10×His-hAQP9 in crude lysates of five selected clones of transformed yeast (CO2, CO8, CO17, WT4, WT22). CO8 is the clone expressing the highest amount of 10×His-hAQP9 protein. The theoretical size of 10×His-hAQP9 is 34.5 kDa. A purified His-tagged spinach aquaporin, SoPIP2;1, is used as positive control and standard. The expected size of monomeric and multimeric forms of SoPIP2;1 are indicated. Lanes have been deleted to position the SoPIP2;1 standard next to the samples. CO, codon optimized; WT, wild type hAQP9 gene.

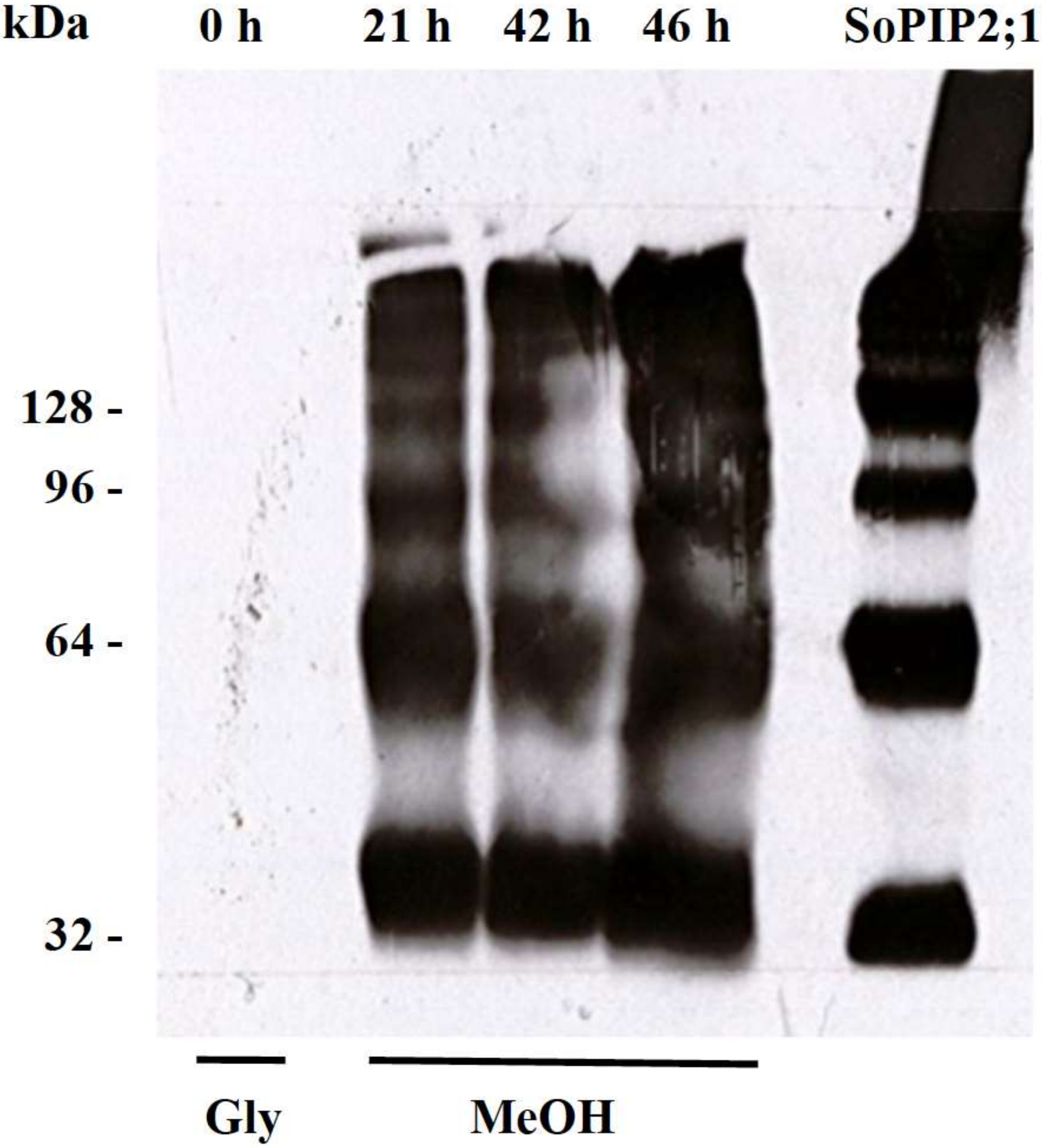

Figure S2. Time course of 10×His-hAQP9 expression. Western blot of crude lysates of samples collected before induction and at indicated time points during methanol induction of expression 10×His-hAQP9, using clone CO8 in a feed-batch fermentor. His-tagged SoPIP2;1 was loaded as a positive control and standard. The expected size of monomeric and multimeric forms of SoPIP2:1 are indicated. The theoretical size of 10×His-hAQP9 is 34.5 kDa. Gly, glycerol feed; MeOH, methanol feed. .

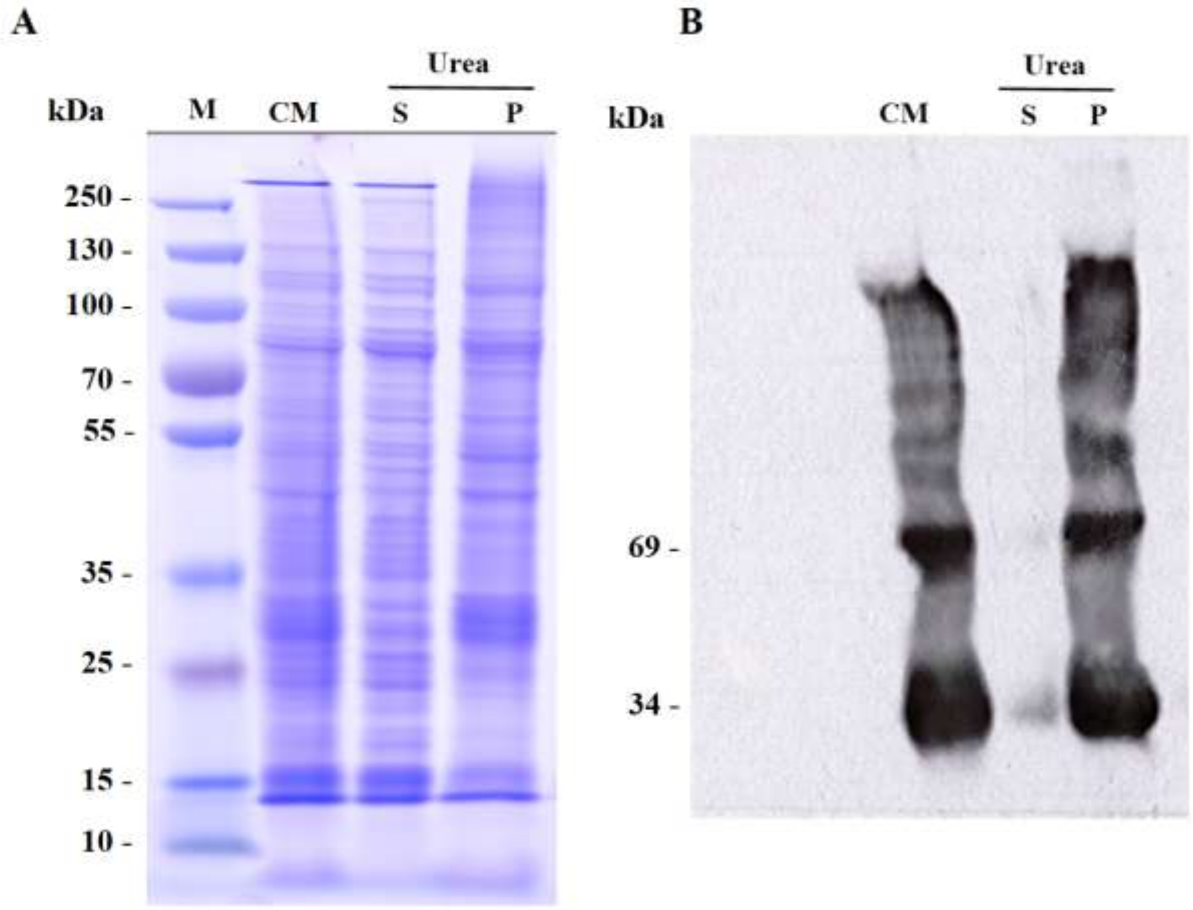

Figure S3. Crude membranes containing 10×His-hAQP9 washed with urea. Crude membranes (CM) isolated from the P. pastoris clone CO8 expressing 10×His-hAQP9 were washed with a buffer containg 4 M urea to remove peripheral membrane proteins. Equal amounts of total protein of the pellet (P) and the supernatant (S) after the wash, as well as of the crude membranes were compared by SDS-PAGE after Coomassie staining (A) and by Western blot using a His-tag antibody (B). The expected size of the monomer and the dimer of 10×His-hAQP9 are 34.5 and 69 kDa, respectively. M, protein size marker.

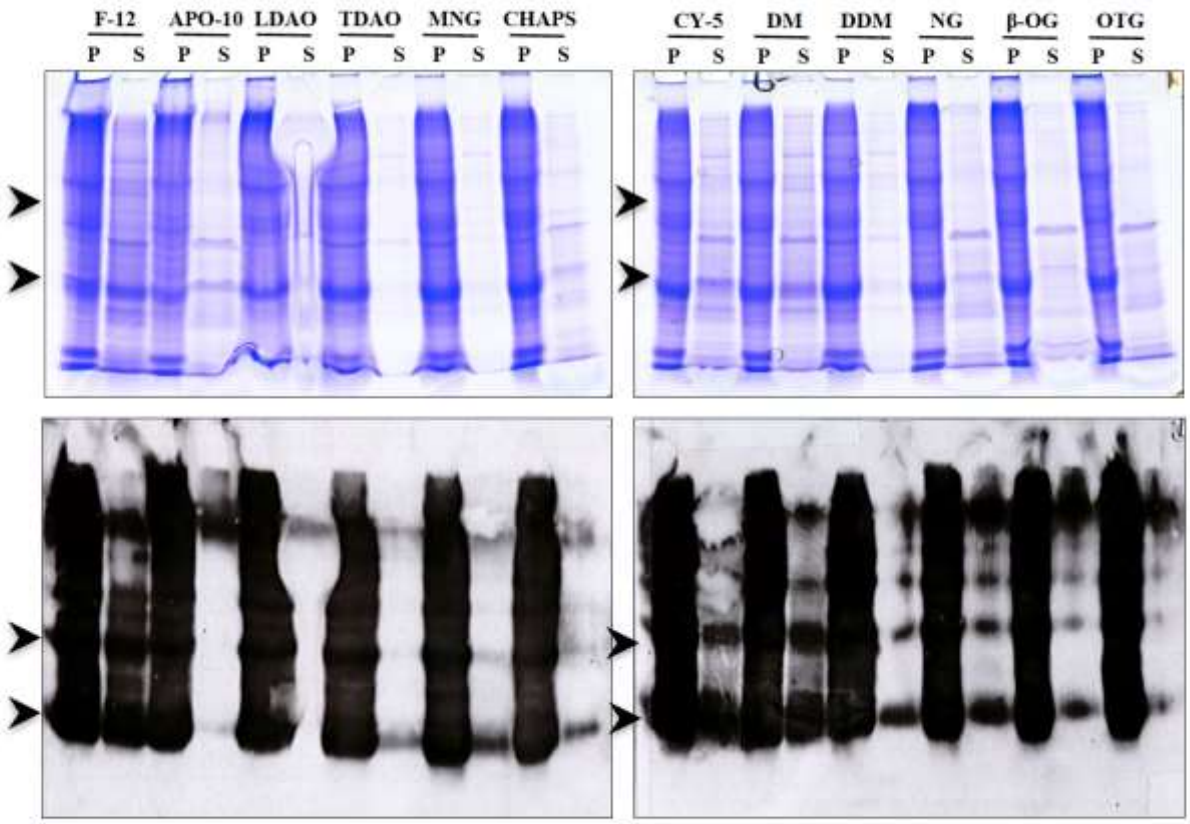

Figure S4. Detergent screening. Washed membranes were solubilized with different detergents and the relative efficacy was evaluated by comparing the resulting pellets (P) and supernatants (S) on SDS-PAGE after Coomassie staining (upper panels) and by Western blot using a His-tag antibody (lower panels). Apart from the harsh zwitter ionic detergent FOS--choline--12 (F-12), n-decyl-ß-D-maltopyranoside (DM) is the most efficient of the tested detergents in the solubilization trials of 10×His-hAQP9. Approximate position of the monomer and dimer of 10×His-hAQP9 is indicated by arrowheads. Dimethyl-decylphosphine oxide (APO-10), lauryldimethylamine N-oxide (LDAO), n-Tetradecyl-N,N- dimethylamine-N-oxide (TDAO), maltose-neopentyl glycol (MNG), 3-[(3-chol-amidopropyl) dimethylammonio]-1-propanesulfonate (CHAPS), 5-Cyclohexyl-1-penty l-β-d-maltoside (CY-5), n--Dodecyl--β--maltoside (DDM), n-nonyl-β-d-glucopyranoside (NG), Octyl--β--glucopyranoside (β-OG), n--Octyl--1--thio--β--D--glucopyranoside (OTG).

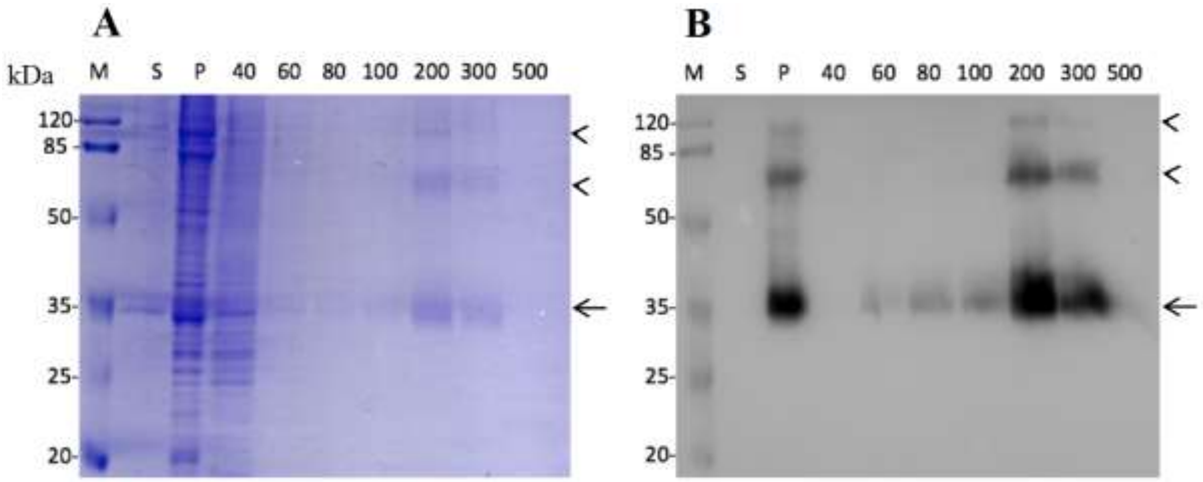

Figure S5. Purification of the 10×His-hAQP9 protein. Purification of 10×His-hAQP9 protein (single arrows: monomeric form; arrowheads: multimeric forms) was accomplished using Ni-NTA affinity chromatography by gravity flow and eluting with growing concentrations of imidazole (40-500 mM). Samples were separated by SDS-PAGE and analyzed by Coomassie blue staining (A) or immunoblotting using an anti-10×His tag polyclonal antibody (B). Binding of 10×His-hAQP9 to the Ni-NTA matrix was relatively strong. Poor elution was seen below 200 mM imidazole while bound 10×His-hAQP9 was completely eluted between 200 and 300 mM imidazole. Samples of supernatant (S) and pellet (P) of obtained with the washed membranes from the P. pastoris (clone CO8) expressing 10×His-hAQP9 were used as control. M, marker of molecular weight.
